# An Analysis of the Clinical and Radiological Prognostic Factors Affecting the Outcomes of Lumbar Intradiscal Biacuplasty

**DOI:** 10.7150/ijms.85777

**Published:** 2023-07-09

**Authors:** Meng-Yin Yang, Chin-Hwee Goh, Chin-Ying Wu, You-Pen Chiu, Hui-Ru Ji, I-Han Hsiao, Cheng-Di Chiu

**Affiliations:** 1 Department of Neurosurgery, Neurological Institute, Taichung Veterans General Hospital, Taichung, Taiwan.; 2 Graduate Institute of Medical Sciences, National Defense Medical Center, Taipei, Taiwan.; 3 College of Nursing, Central Taiwan University of Science and Technology, Taichung, Taiwan.; 4 Department of Post-Baccalaureate Medicine, College of Medicine, National Chung Hsing University, Taichung, Taiwan.; 5 Department of Neurosurgery, Sarawak General Hospial, Kuching, Sarawak, East Malaysia.; 6 Department of Neurosurgery, China Medical University Hsinchu Hospital, Hsinchu, Taiwan.; 7 Graduate Institute of Integrated Medicine, China Medical University, Taichung, Taiwan.; 8 Department of Neurosurgery, China Medical University Hospital, Taichung, Taiwan.; 9 Graduate Institute of Biomedical Science, China Medical University, Taichung, Taiwan.; 10 School of Medicine, China Medical University, Taichung, Taiwan.; 11 Spine Center, China Medical University Hospital, Taichung, Taiwan.

**Keywords:** intradiscal biacuplasty, disc degenerative disease, herniated intervertebral disc, lumbar magnetic resonance imaging, prognostic factor

## Abstract

**Purpose:** Intradiscal biacuplasty (IDB) has been proven to be effective for treating lumbar degenerative disc disease (DDD). However, there has not been a reported prognostic factor for IDB. The present study meticulously evaluates the general and radiographic features that may serve as markers for predicting the therapeutic outcome of IDB.

**Methods:** A prospective case series study was conducted, following time-series analysis moving averages models, with forty-one patients suffering from chronic discogenic lower back pain for more than six months. These patients subsequently received lumbar cool radiofrequency IDB and were enrolled in the study. Thirty-seven patients completed follow-up questionnaires at 1, 3, 6, and 12 months. The surgical outcomes were reported using visual analogue scale (VAS), Oswestry disability index (ODI), and the consumption of nonsteroidal anti-inflammatory drugs (NSAID). Furthermore, a univariate analysis was performed to identify prognostic factors associated with pain relief from age, gender, body mass index (BMI), and pre-operative lumbar magnetic resonance imaging reading.

**Results**: Significant reductions were found in estimated VAS and ODI at the post-operative period at 1, 3, 6, and 12 months (*P* < 0.001). The NSAID dosage was significantly decreased at 3-month and 1-year follow-up (*P* < 0.05). No procedure-related complications were detected. The prognosis of IDB was not related to disc height, Pfirrmann grading or Modic endplate change. However, disc extrusions were associated with promising outcomes (VAS improvement ≥ 50%) on pain relief (*P* < 0.05).

**Conclusion:** IDB is a good alternative choice for treating lumbar DDD. Patients with a painful extrusion lumbar disc may gain some benefits after receiving IDB following a period of failed conservative treatment. These findings may also add some references for physicians in the decision making when treating lumbar DDD.

## Introduction

Lumbar degenerative disc disease (DDD) is a multifactorial progressive disease which may stem from genetic, metabolic, biomechanics, biochemical, environmental, and individual adverse risk factors 1-3. DDD is believed to be one of the major origins of lower back pain; however, its definition and diagnosis still lack uniformity 4. Most physicians agree that the typical symptoms of DDD consist of chronic axial lower back pain when sitting or bending forward. The classic lumbar magnetic resonance imaging (L-MRI) characteristics of DDD may include disc dehydration (black disc), internal annulus rupture, disc prolapse, and the presence of a high-intensity zone (HIZ) 5, 6. In addition, lumbar challenging discography as a diagnostic test is still equivocal 7. Though most DDD are asymptomatic, some stepwise therapeutic treatments focus on the symptomatic ones according to the severity level. Medication, rehabilitation, interventional therapy, minimally invasive surgery, and surgical treatment have been used to cure or relieve the pain of these intradiscal disorders 8.

Among all the interventional therapies, intradiscal biacuplasty (IDB, Baylis Medical Co., Montreal, Canada) is one of the thermal annular procedures (TAPs), approved by the United States Food and Drug Administration in 2005 9. Since then, evidence-based documentation has recommended IDB as a therapeutic choice for chronic, refractory discogenic pain 10. The mechanism of TAPs is the generation of sufficient annular temperatures that leads to denervation and pain relief 11. Intradiscal electrothermal therapy (IDET, Smith and Nephews, London, UK), approved by the United States Food and Drug Administration in 1998 12, is another forerunner of TAP. The IDET utilizes a flexible active-tip intradiscal catheter placed in the junction between the annulus and nucleus pulposus to elicit electrothermal therapy 13. However, this long, flexible coil catheter is difficult to place in the proper place, resulting in debatable therapeutic results 14. Even a randomized, double-blind controlled study found that the IDET gained no significant benefit over placebo 15. In contrast, IDB places two radiofrequency probes under fluoroscopic guidance, which is a much easier procedure and has fewer application-related complications than IDET 16. According to a systematic review investigating the effectiveness of TAPs, IDB was put on a higher level of evidence (level I, strong) than IDET (level III, fair) for the treatment of chronic, refractory discogenic pain 17.

To our knowledge, there is no prognostic assessment of IDB according to the patients' characteristics and image findings. In the present prospective study, we analyzed clinical data findings based on 41 patients who received IDB by one surgeon in a single medical center, with the hope of providing a reference to physicians who want to perform this procedure.

## Materials and methods

### Eligibility and patient enrollment

**Figure 1:**
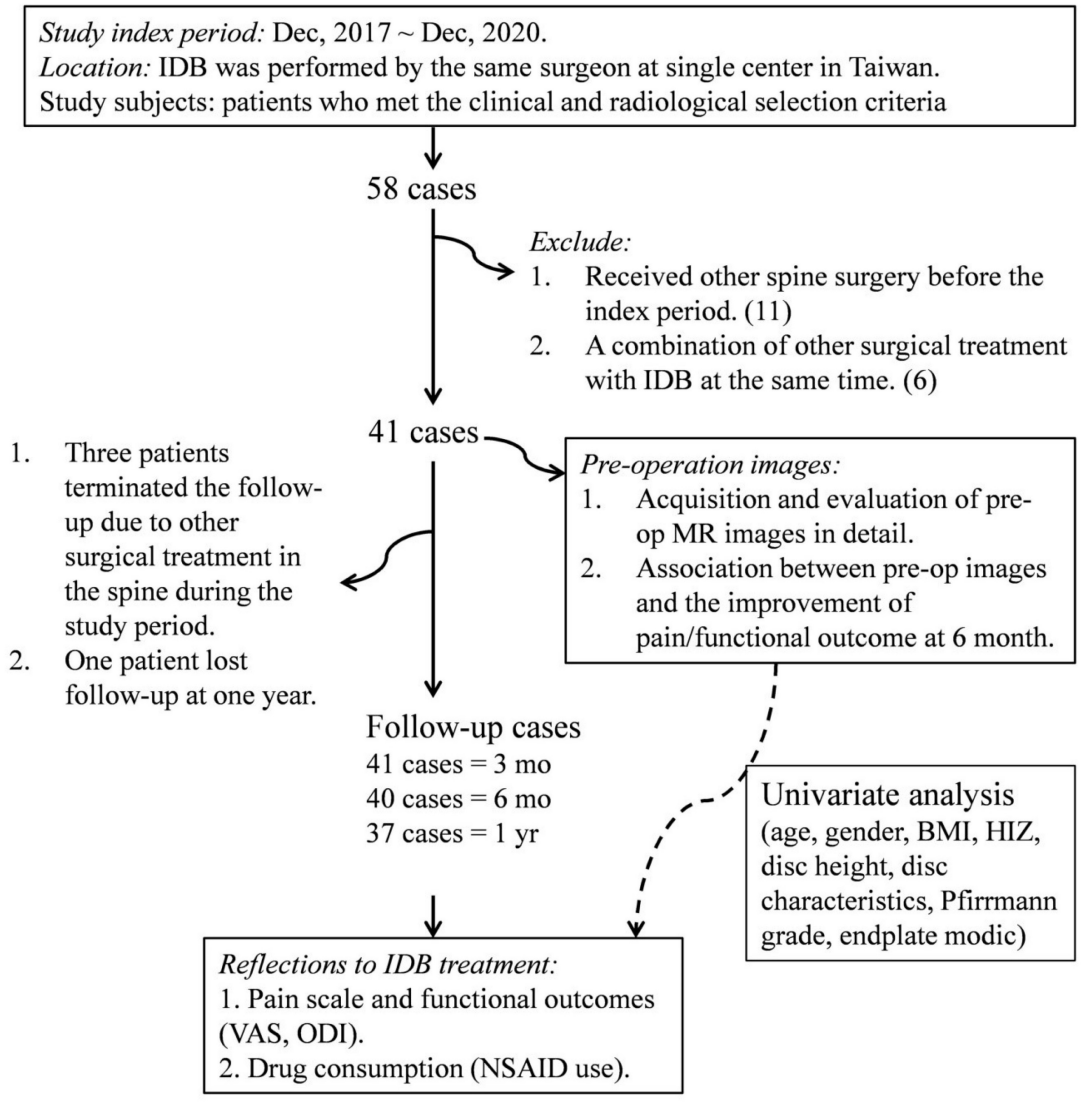
** A flow diagram showing patient enrollment, data collection, and data analysis of the study.** IDB: intradiscal biacuplasty; DDD: degenerative disc disease; MRI: magnetic resonance imaging; VAS: visual analogue scale; ODI: Oswestry disability index; NSAIDs: nonsteroidal anti-inflammatory drugs; BMI: body mass index; HIZ: high intensity zone.

The ethical approval of the present study was obtained from the research ethics committee of China Medical University Hospital (CMUH104-REC3-091). All methods were performed in accordance with the relevant guidelines and regulations. We started a prospective case series study following time-series analysis moving averages models from December 2017 to December 2020, a total of 58 IDB procedures with 74 discs were performed by one physician in the neurosurgical department of CMUH **(Figure 1)**. The study selection criteria were: 1) patients with a history of chronic lower back pain that is unresponsive to conservative care for a period longer than 6 months; 2) Age > 20 years; 3) disc height preservation > 50% compared to the adjacent disc; 4) presenting with axial back pain more frequently than leg pain, which was exacerbated by back flexion; 5) evidence of disc degeneration at ≤ three levels (Pfirrmann grade II-V) on L-MRI within 3 months; 6) the concordant degenerative disc showed positive reproducible pain provoked by intraoperative challenge discography. We diagnosed putatively painful discs according to several diagnostic properties detected on pre-operative L-MRI (herniated discs with characteristic degenerative changes) that were compatible with the patient's clinical signs indicating discogenic pain (axial pain exacerbated by bending forward at the back). We attempted intraoperative challenge discography on all suspected disc levels identified by both clinical and imaging findings. IDB treatment was applied to multiple disc levels simultaneously if reproducible pain appeared in multiple levels. By contrast, we abandoned the surgical treatment in cases where the challenge discography was not responsive. After excluding candidates with a previous history of tumors, unexplained bleeding/infection at the anticipated needle entry site, a history of opioid abuse, or existing herniated disc fragment in L-MRI, forty-one patients were enrolled as study subjects. Prior to entering the study, all patients had agreed and signed an informed consent form with a clear comprehension of the study details. All patients completed the 3-month follow-up outcome assessment questionnaire, whereas only 37 had completed the 12-month follow-up questionnaire except one lost follow-up at one year and 3 received a second surgery at 4, 8, and 9 months, respectively. Their follow-ups were terminated when they received the second surgery. Several long-term surveys were conducted via phone interview due to the geographical location. Drug consumption data were collected for 31 cases, taking into consideration the acceptance of prescribed nonsteroidal anti-inflammatory drugs (NSAID) at any follow-up period.

### Procedure of IDB

Prophylactic antibiotic was administered 30 minutes prior to the procedure. The patients were in a prone position with pillow support and ankle pads for comfort. Patients then received local anesthesia or intravenous sedation, including fentanyl, propofol or midazolam. Two transdiscal introducers were used through a posterolateral, oblique approach under fluoroscopic guidance to gain access to intervertebral discs. The provocative challenge discography was then performed to confirm whether pain was reproducible in the concordant disc level. Next, radiofrequency probes were positioned in the posterior 1/3 of the disc in the lateral C-arm view to access the proper positions over the annulus-nucleus junction of both probes. Our heating protocol increased the temperature to 50 °C gradually over a 15-minute period.

### Outcome assessment

At 1, 3, 6, and 12 months after surgery, patients in the outpatient clinic were asked to mark their pain level using a 10-cm visual analogue scale (VAS) 18. Additionally, the physical disability resulting from the degenerative discs was determined using the Oswestry Disability Index (ODI) composed of a 10-item, 50-point questionnaire 19. To get an accurate value of the effect size, the post-operative improvement of VAS and ODI were calculated by dividing the difference between the means of the baseline and 6-month values. The daily use of NSAIDs was also recorded without discerning between single or multiple dispensations. The dosage of the multiple NSAID dispensation was taken into account.

### General and image variables

Several variables were used in the present study to study their association to the improvement of pain relief. Age, gender, and body mass index (BMI) of patients were obtained from general clinical data. Patients with a BMI ≤ 25 were considered to be “normal” whereas a BMI > 25 was defined as “overweight.” Variables from spinal characteristics, including the maintained disc height (75-100% or 50-75% preservation), HIZ, endplate Modic (Type I and Type II), Pfirrmann grading (I-V), and types of disc herniation (protrusion and extrusion), were estimated from pre-operative L-MRI. Treated disc with a Pfirrmann grading I-III was regarded as “mild” degeneration whereas IV-V was deemed as “severe” degeneration. Changes in endplate Modic were defined as the existence of both types of changes.

### Statistical analysis

One-way analysis of variance with Turkey HSD post-hoc comparisons was used to analyze the between-group difference of VAS, ODI, and NSAID use at different follow-up time. The effects of pre-operative types of disc herniation on VAS or ODI improvement were determined using Mann-Whitney U-test. Moreover, univariate analysis of general and image variables was done using the Chi-Square test where a VAS improvement equal to or over 50% was considered to be effective in treatment. The mean differences in VAS improvement of each variable were determined by Student's *t*-test. Analytic results with a *P*-value < 0.05 were regarded as statistically significant. All analyses were performed using GraphPad Prism 7 (GraphPad Software, Inc., CA, US).

## Results

**Figure 2:**
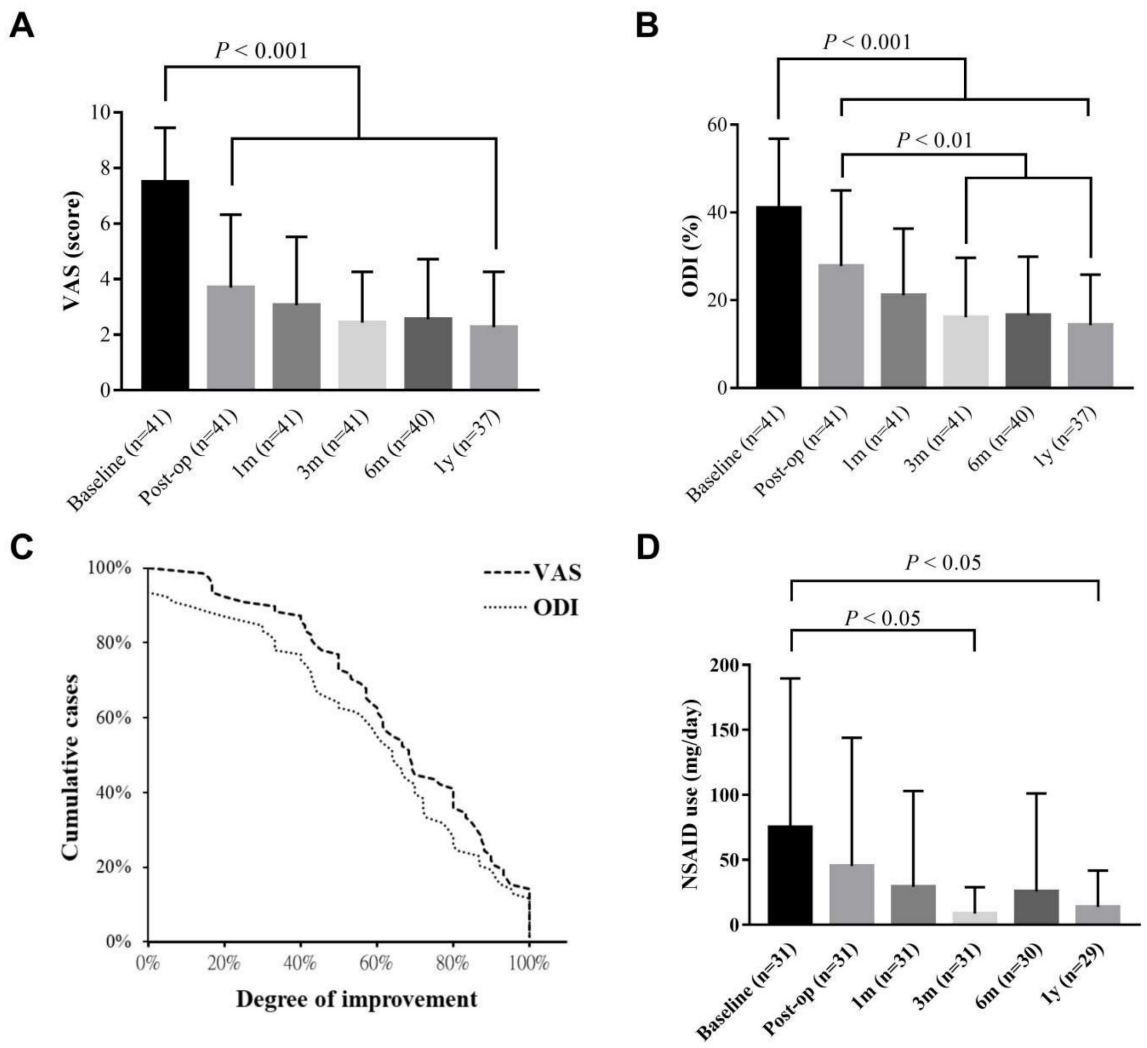
** Long-term follow-up of pain/functional scales and drug consumption.** The chart showing the estimated VAS (a) and ODI (b) from last post-operative record (baseline) to that of one-year follow-up. (c) Improvement of both scales was determined by the records of baseline and 6-month follow-up. (d) The consumption of NSAIDs. All data are presented as mean ± SD. VAS: visual analogue scale; ODI: Oswestry disability index; NSAIDs: nonsteroidal anti-inflammatory drugs.

**Figure 1** shows the detailed protocol for the patients' inclusion, exclusion, and data mining. Clinical data from 41 patients with an average age of 49.44 ± 10.78 (23-74 years old) were entered into the final evaluation (**Table 1**). The average follow-up period was 714.95 ± 243.05 days, during which no known serious adverse event occurred. Significant improvement was found on both VAS and ODI in response to IDB treatment (*P* < 0.001, **Figure 2**). Post-operatively, the Mean VAS was changed from 7.49 ± 1.95 (baseline) to 3.70 ± 2.62, and to 2.44 ± 1.82 at 6 month. The reductions were stable and maintained at a 1-year follow-up. Similar effects were also be observed on the ODI score, which was changed from the baseline value of 41.02 ± 15.80 to 27.76 ± 17.28 at post-operation. Moreover, the value gradually reduced from the 3-month to the one-year follow-up (*P* < 0.01). Based on the findings, most patients (33/40, 82.5%) had at least 50% VAS improvement at the 6 month. However, a re-surgery rate of 7.3% (3/41) was observed with the follow-up period. One patient received percutaneous endoscopic lumbar discotomy at L4/5 8 months after two-level IDB treatment (L4/5 and L5/S1) due to persistent pain, and another patient received fusion surgery 4 months after IDB treatment in the same levels (L4/5) due to intolerable back pain. The use of NSAIDs was also significantly reduced at 3 and 6 months (*P* < 0.05, **Figure 2d**). The average free-to-NSAIDs-use period was 137.19 ± 144.95 days.

**Figure 3:**
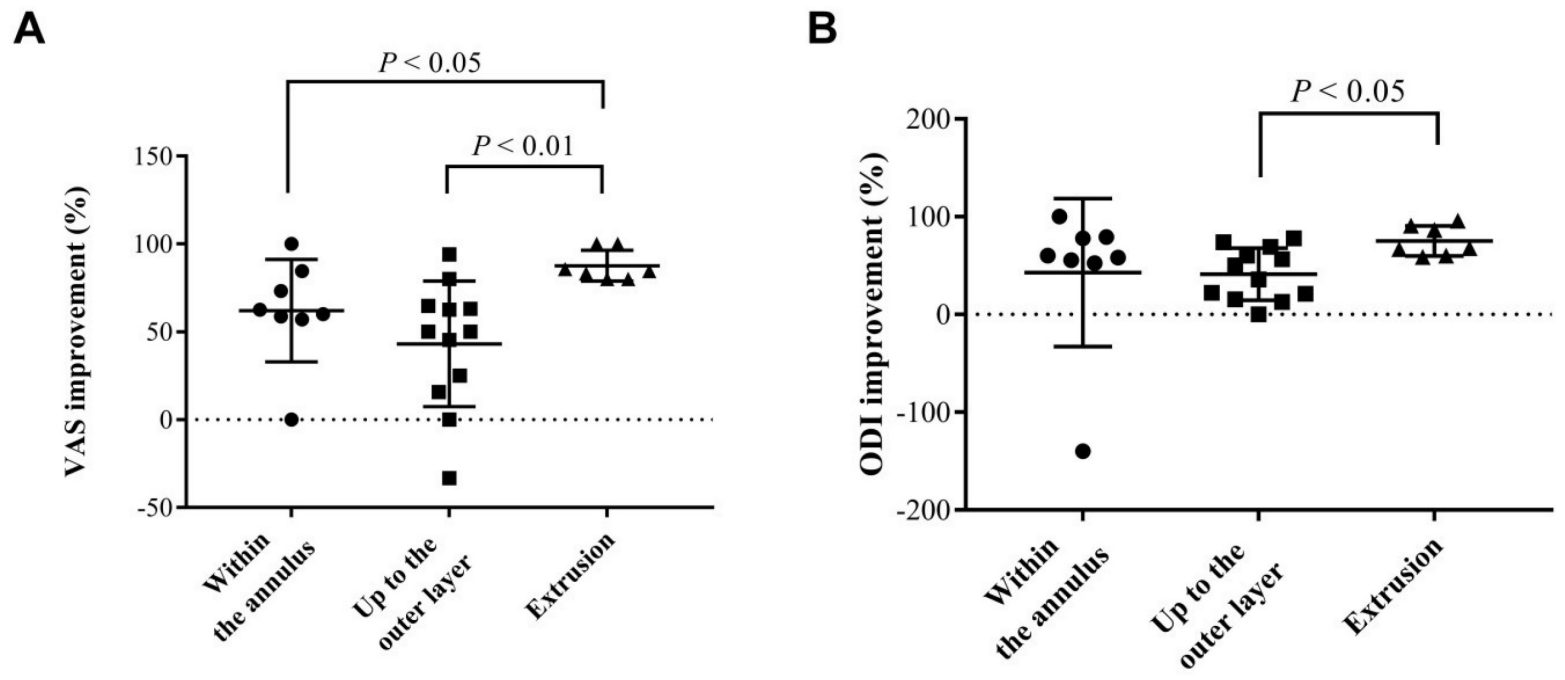
** Analysis of the influences of different types of disc herniation on estimated pain/functional outcomes after IDB treatment.** The mean 6-month improvements of (a) VAS and (b) ODI from each group were determined. The characteristics of disc herniation were classified into three groups, including two types of contained discs (within the annulus and up to the outer layer of the annulus) and extruded discs. VAS: visual analogue scale; ODI: Oswestry disability index.

Univariate analysis of variables from general and image data was performed to determine critical factors for the prediction of positive response to IDB on substantial VAS improvement (**Table 2**). No association was found in basic information, including BMI, patient's age, and sex. For image characteristics, neither the presence of HIZ, Pfirrmann disc degeneration grading, the Modic changes in endplate, and disc height had no statistical significance. However, with respect to the characteristics of disc herniation, the result showed that disc protrusion was associated with significantly poor, substantial pain relief (OR: 0.10, *P* < 0.05). Furthermore, we analyzed the improvement of pain and functional scales in detail characteristics based on the degree of extension of the herniated discs (**Figure 3**). There was no sequestrated disc in our study. In comparison to disc extrusion, disc protrusion within the annulus had less VAS and ODI improvement after IDB treatment (*P* < 0.05). The results indicated that for IDB more optimistic prognosis in pain relief can be found in patients with a disc extrusion (herniation beyond the annulus) in comparison with those with a disc protrusion.

## Discussion

According to the present data, we echo that IDB is one of the most effective TAPs for the treatment of DDD as most patients can achieve not only significant pain relief (33/40 patients with ≥ 50% VAS improvement) but also improved quality of life (31/40 patients with a ≥ 10-point decrease of ODI) within 6 months of post-surgery 20, 21. Furthermore, the efficacy can continue up to a year (**Figure 2**). The cost of IDB is not covered by National Health Insurance in Taiwan. However, in comparison to open surgical procedure which is paid by National Health Insurance, IDB is an outpatient percutaneous procedure that accesses minimally invasive and has a lower rate of adverse events 22. The advantages of IDB originated from its ability to modify annular collagen and to ablate sensitized nociceptors, distributing around the annular-nuclear junction of the intervertebral disc 9. In addition, the design of cool and bipolar radiofrequency encompassed a larger therapeutic area in a safer manner under a lower temperature, superior to conventional RF 17. The therapeutic value of IDB in DDD has also been validated by two high-quality randomized control trials 23, 24.

According to the publications concerning TAPs, the criteria for patient enrollment is quite diverse, which may contribute to the lack of unified prognostic factors 25. For example, it is unclear what is the upper age boundary of the therapeutic. Some groups limit the patients' age below 55 years old, and some below 60 16, 26. Regarding IDB, whether the age factor impacts the therapeutic outcome has not been addressed. According to the present results, there is no significant difference in effectiveness between the < 55 and the > 55 age groups (**Table 2**). We suppose that the therapeutic age limitation of IDB can be higher than nucleoplasty (NP) based on two possible reasons: (1) IDB mainly ablates sensitized nociceptors in lower temperature (50 °C) and longer duration (15 minutes), age is less of a factor. However, NP coblates the nucleus pulpous beyond 70 °C which intends to reduce the disc volume and intradiscal pressure, both of which are dependent on a younger age factor 27. (2) The distinct targeting region of a disc is different. The IDB works around the nucleus-annulus junction to modify annular collagen whereas NP targets the center of the nucleus which is dependent on a young, water-rich disc 11. These two crucial points may relate to Kapural et al. who had reported a successful treatment of a discectomized disc via IDB 28. In addition, our study also showed there was no significant therapeutic impact for IDB in gender and BMI (**Table 2**).

According to the enrollment of 41 patients in the present study, the majority of them (63.4%) are below 55 years old. However, the most common causes of lumbar DDD in youger patients result from overweight, trauma, overuse, or improper sports-related injuries 29. Many of these patients experienced improvement after reducing their body weight, preventing trauma, and engaging in effective athletic training and preparation programs 30. Furthermore, in order to prevent sports-related lower back pain, which often serves as a warning sign of lumbar DDD, Farì et al. recommended implementing appropriate prevention strategies within a comprehensive rehabilitation program to optimize the health benefits 31. If back pain persists and is clinically consistent with lumbar DDD, and L-MRI reveals extruded lumbar disc, it is advisable to consider IDB after attempting conservative rehabilitation treatment to minimize disc damage.

Apart from patients' characteristics, it has yet to determine whether the pre-operative radiological characteristics affect the clinical outcomes of IDB. In the present study based on the pre-operative L-MRI, we show that the clinical outcomes can be affected by the characteristics (protrusion or extrusion) of the herniation of the intervertebral disc. Jae Chul Lee et al. also found similar results in conventional lumbar open discectomy 32. Because an adequate evaluation of the prognostic factors is important for the accurate determination of the surgical indications, the present study may help position IDB as one of the stepwise strategies in DDD treatment. The IDB is more optimistic for pain relief in patients with a disc extrusion (herniation beyond the annulus) than those with a disc protrusion. Thus, conservative treatment should be the first consideration for patients with a bulging disc where the nucleus of a spinal disc remains contained within the annulus fibrosus. In addition, good patient selection for interventional intradiscal therapy may help to postpone the irreversible spine fusion surgery 8, 33.

Under our patient selection criteria, three patients required a second surgery after IDB treatment due to persistent back pain. One possible explanation is that discal nerve regeneration may result in the reconstruction of a nociceptive pathway that was previously blocked by IDB 21, 23, 34. However, the underlying pathogenesis leading to persistent pain and the timing of this phenomenon remain unclear. Another possible explanation involves false-positive discography reports concealed by the use of painkillers, individual pain responses, or emotional issues 35. Inappropriate postures, aggressive exercise, or repeated trauma may also lead to reinjuries, resulting in persistent low-back pain.

The study design has many limitations, such as the lack of randomization and a control group, a small number of cases, and a short follow-up period. In addition, the results still lack long-term evaluation of the prognostic factors. Another issue is that protruded discs have higher rate of spontaneous regression than extruded discs under conservative care, which may result in favorable outcomes 36. A large randomized controlled trial is essential to further investigate whether or when IDB should be done for the optimal treatment of different types of herniated discs. However, the present study preliminary shows the clinical results of 41 cases (27 with single-level treatment) receiving IDB by a single surgeon. To our knowledge, this is the first case of an outcome prediction analysis of clinical and radiographic prognostic factors of IDB.

## Conclusion

Although there has not been an accurate method to predict the outcomes of IDB for painful lumbar disorder, signs on L-MRI such as disc extrusion may be an available marker for predicting a good prognosis.

## Figures and Tables

**Table 1:** Demographic data

Subjects (n = 41)	DATA Event ^a^
Age (yrs)	49.44 ± 10.78
> 55	15
≤ 55	26
Body mass index	26.10 ± 3.39
Gender (F/M)	15/26
Level treated (1/2/3 level) ^b^	27/12/2
Total disc spaces (segments)	57
L2/3	2
L3/4	6
L4/5	31
L5/S1	18
Baseline VAS ^c^	7.49 ± 1.95
Baseline ODI	41.02 ± 15.80
Baseline NSAIDs use (mg/day)^ d^	75.00 ± 114.56

a Data was presented as mean ± SD b The sum of treated disc in an IDB surgery. c The baseline values of VAS/ODI were obtained from the latest recorded survey before the operation d Include pan NSAID drugs or any compounded medicine. VAS: visual analogue scale; ODI: Oswestry disability index; NSAIDs: nonsteroidal anti-inflammatory drugs.

**Table 2:** Univariate analysis of clinical variables associated with prognostic outcomes

Variables	VAS^ a^			
	Mean ± SD (%) (N)	*P* value^ b^	OR (95% Cl)^ c^	*P* value
BMI (normal vs. overweight)	65.30± 31.53 (18) vs. 62.24 ± 32.85 (22)	.919	1.63 (0.39-6.81)	.499
Age (≤55y vs. >55y)	63.14 ± 28.82 (26) vs. 67.65 ± 37.86 (15)	.675	0.61 (0.13-2.81)	.528
Gender (F vs. M)	61.75 ± 37.21 (14) vs. 66.31 ± 29.25 (26)	.672	1.63 (0.35-7.48)	.528
**Image characteristics (n = 27)** ^d^				
HIZ (no vs. yes)	61.47 ± 25.30 (12) vs. 59.33 ± 39.86 (15)	.873	0.73 (0.14-3.82)	.706
Disc height (>75% vs. 50-75%)	62.11 ± 33.60 (23) vs. 49.72 ± 36.28 (4)	.506	2.83 (0.32-24.81)	.334
Characteristics (protrusion vs. extrusion)	**50.69 ± 33.85 (20) vs. 87.67 ± 8.70 (7)**	.009*	**0.10 (0.01-1.95)**	.046*
Pfirrmann grade (mild vs. severe)	66.89 ± 32.51 (10) vs. 56.39 ± 34.56 (17)	.443	2.18 (0.35-13.76)	.401
Endplate Modic (w/o change vs. changes)	57.38 ± 34.57 (24) vs. 83.44 ± 3.03 (3)	.211	0.28 (0.01-6.01)	.233

a Six-month VAS improvement. b Between-group analysis conducted using Student T-test. c Odd's ratios were acquired by Chi-square analysis of > 50% VAS improvement. d Image data from patients who received multiple level treatments were not included in this analysis. VAS: visual analogue scale; BMI: body mass index; HIZ: high-intensity zone.

## Abbreviations

DDD: degenerative disc disease
L-MRI: lumbar magnetic resonance imaging
HIZ: high-intensity zone
IDB: intradiscal biacuplasty
TAPs: thermal annular procedures
IDET: Intradiscal electrothermal therapy
NSAID: nonsteroidal anti-inflammatory drugs
VAS: visual analogue scale
ODI: visual analogue scale
BMI: body mass index
NP: nucleoplasty
